# Knockdown of *SPRY4* and *SPRY4-IT1* inhibits cell growth and phosphorylation of Akt in human testicular germ cell tumours

**DOI:** 10.1038/s41598-018-20846-8

**Published:** 2018-02-06

**Authors:** Mrinal K. Das, Kari Furu, Herman F. Evensen, Øyvind P. Haugen, Trine B. Haugen

**Affiliations:** 1 Faculty of Health Sciences, OsloMet — Oslo Metropolitan University, Oslo, Norway; 2 Cancer Registry, Oslo, Norway; 3 Faculty of Dentistry, University of Oslo, Oslo, Norway

## Abstract

Testicular germ cell tumour (TGCT) is the most common cancer in young men in large parts of the world, but the aetiology is mainly unknown. Genome-wide association studies have so far identified about 50 susceptibility loci associated with TGCT, including *SPRY4*. SPRY4 has shown tumour suppressor activity in several cancer cells, such as lung and prostate, while it was found to act as an oncogene in ovarian cancer. An intronic region within the *SPRY4* gene produces a long non-coding RNA, *SPRY4-IT1*, which has been reported to act as an oncogene in melanoma, breast cancer, and colorectal cancer, and as a tumour suppressor in lung cancer. The roles of *SPRY4* and *SPRY4-IT1* in TGCT development are yet unknown. We found higher expression levels of *SPRY4*, both mRNA and protein, and of *SPRY4-IT1* in human TGCT than in normal adult testis. Small-interfering RNA (siRNA)-mediated transient knockdown of *SPRY4* and *SPRY4-IT1* in two TGCT cell lines 833 K and NT2-D1 resulted in decreased cell growth, migration, and invasion. Knockdown of *SPRY4* and *SPRY4-IT1* also led to a significant reduction in the phosphorylation of Akt. Our findings indicate that *SPRY4* and *SPRY4-IT1* may act as oncogenes in TGCTs via activation of the PI3K / Akt signalling pathway.

## Introduction

Testicular germ cell tumour (TGCT) is the most common malignancy in young men in large parts of the world. The incidence of TGCT has been increasing over the last decades and is highest in white Caucasian populations in industrialised countries and lowest in men of African ancestry^1–3^. The aetiology of TGCT is poorly understood. Both genetic and environmental factors are believed to contribute to the disease risk^4,5^, of which approximately 25% of TGCT susceptibility may be caused by genetic effects^6,7^. The precursor cell to TGCT, carcinoma *in situ*, resembles primordial germ cells, and there is epidemiological evidence indicating that TGCT originates in fetal life^8,9^. However, some studies suggest that environmental exposures in adolescence and adulthood are also associated with TGCT risk^4,10^.

Genome-wide association (GWA) studies of TGCT have so far identified about 50 susceptibility loci^11–13^, and *SPRY4* is one of the genes, which shows strong and consistent association^9,13–15^. *SPRY4* belongs to a family of four genes (*SPRY1–4*) encoding proteins which are well-known regulators of receptor tyrosine kinases (RTKs)^16^. The RTK-mediated MAPK / ERK and PI3K / Akt signalling pathways are involved in the homeostasis of cell growth and differentiation, and in cancer, activation of these pathways leads to increased cell proliferation, survival, invasion, and metastasis^17^. Altered expression of *SPRY* in cancer may cause aberrant regulation of MAPK / ERK and PI3 / Akt signalling pathways^18^. Involvement of *SPRY4* in the regulation of tumorigenesis has already been documented in several human cancer types^18^. In lung and prostate cancer, SPRY4 showed tumour suppressor activity^19,20^, whereas in ovarian cancer, knockdown of *SPRY4* attenuated growth factor-induced cancer progression^21^. SPRY4 may also play a role in the regulation of cell growth and differentiation in TGCT pathogenesis. Moreover, *SPRY4-IT1*, a long non-coding RNA (lncRNA) produced within an intronic region of *SPRY4*, has been reported to promote cancer development in melanoma^22^, breast cancer^23^, and colorectal cancer^24^ while inhibiting lung cancer growth^25^. However, to our knowledge, no effect of *SPRY4-IT1* on RTK-mediated signalling pathways has been reported.

The roles of *SPRY4* and *SPRY4-IT1* in TGCT development are yet unknown. The lack of an appropriate laboratory animal model, as well as the difficulties of establishing primary cultures from human germ cells, make studying human TGCT pathogenesis a challenge. In the present work, we used metastatic TGCT tissue-derived embryonal carcinoma (EC) cell lines 833 K and NT2-D1^26,27^ to explore the roles of *SPRY4* and *SPRY4-IT1*. The effect of siRNA-mediated knockdown on cell growth, migration, and invasion was investigated, as well as on the MAPK / ERK and PI3K / Akt pathways. We also examined the expression of *SPRY4* and *SPRY4-IT1* in several TGCT subtypes and human normal adult testis.

## Results

### Expression of *SPRY4* and *SPRY4-IT1* in TGCT

**Figure 1:**
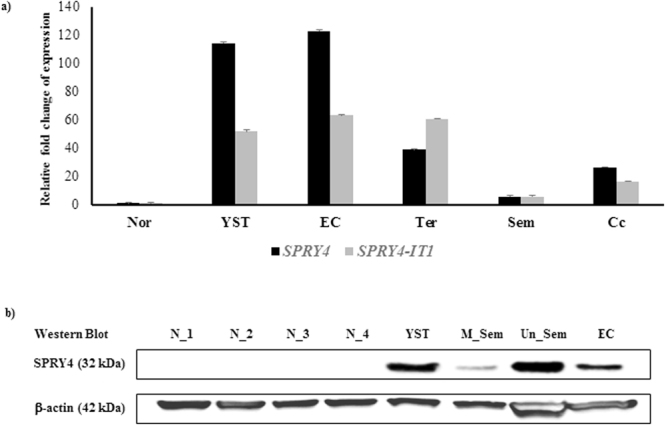
Expression levels of *SPRY4* and *SPRY4-IT1* in TGCT compared to control testis. (**a**) Expression levels of *SPRY4* and *SPRY4-IT1* in TGCT and normal adult testis samples were analysed by qPCR. The relative expression levels of *SPRY4* and *SPRY4-IT1* were significantly higher in all the TGCT samples than in normal samples. Furthermore, the *SPRY4* and *SPRY4-IT1* RNA levels were considerably higher in YST, EC and Ter than in Sem and Cc. There was also a notable difference between *SPRY4* and *SPRY4-IT1* RNA expressions in TGCTs. *SPRY4* expression levels were higher in YST, EC, and Cc than those of *SPRY4-IT1*, whereas *SPRY4-IT1* expression levels were higher in Ter than those of *SPRY4*. Relative fold change of expression was determined using the equation RQ = 2^−ΔΔCT^ where mean dCT value and SD were calculated from each of N (n = 11), YST (n = 3), EC (n = 4), Ter (n = 3), Sem (n = 2) samples except Cc (n = 1). N: Normal; YST: Yolk sac carcinoma; EC: Embryonal carcinoma; Ter: Teratoma; Sem: Seminoma; Cc: Choriocarcinoma. (**b**) Protein levels of SPRY4 in normal and TGCT samples were determined by western blot. No expression of the SPRY4 protein was detected in any normal samples whereas all the TGCT samples showed a detection of SPRY4 protein expression. Furthermore, the band intensity in Un_Sem was much stronger than in M_Sem. β-actin was used as a loading control. The cropped blots are used in the figure, and full-length blots are presented in Supplementary Fig. S1. N (normal); YST (yolk sac tumour); M_Sem (moderately differentiated seminoma); Un_Sem (undifferentiated seminoma); EC (embryonal carcinoma). *t*-test: Normal vs TGCT, mean ± SD, statistical significance p < 0.05.

We examined the expression levels of *SPRY4* and *SPRY4-IT1* in 13 TGCTs and 11 normal testis samples. The TGCT samples comprised of yolk sac tumour (n = 3), embryonal carcinoma (n = 4), teratoma (n = 3), seminoma (n = 2), and choriocarcinoma (n = 1). RNA levels of *SPRY4* and *SPRY4-IT1* were significantly higher in all the TGCT samples than in normal testis samples (Fig. 1a). Furthermore, the *SPRY4* and *SPRY4-IT1* RNA levels in yolk sac tumour, embryonal carcinoma, and teratoma were considerably higher than those in choriocarcinoma and seminoma. There was also a notable difference between *SPRY4* and *SPRY4-IT1* expression patterns in TGCTs. *SPRY4* expression levels were higher than those of *SPRY4-IT1* in yolk sac tumour, embryonal carcinoma, and choriocarcinoma, whereas *SPRY4-IT1* expression levels were higher than those of *SPRY4* in teratoma. SPRY4 protein was also abundantly expressed in all TGCT samples, whereas no expression was detected in any of the normal testis samples (Fig. 1b). A profound difference of SPRY4 protein expression between moderately differentiated seminoma and undifferentiated seminoma was observed, with the highest level in undifferentiated seminoma.

### Knockdown of *SPRY4* and *SPRY4-IT1*

**Figure 2:**
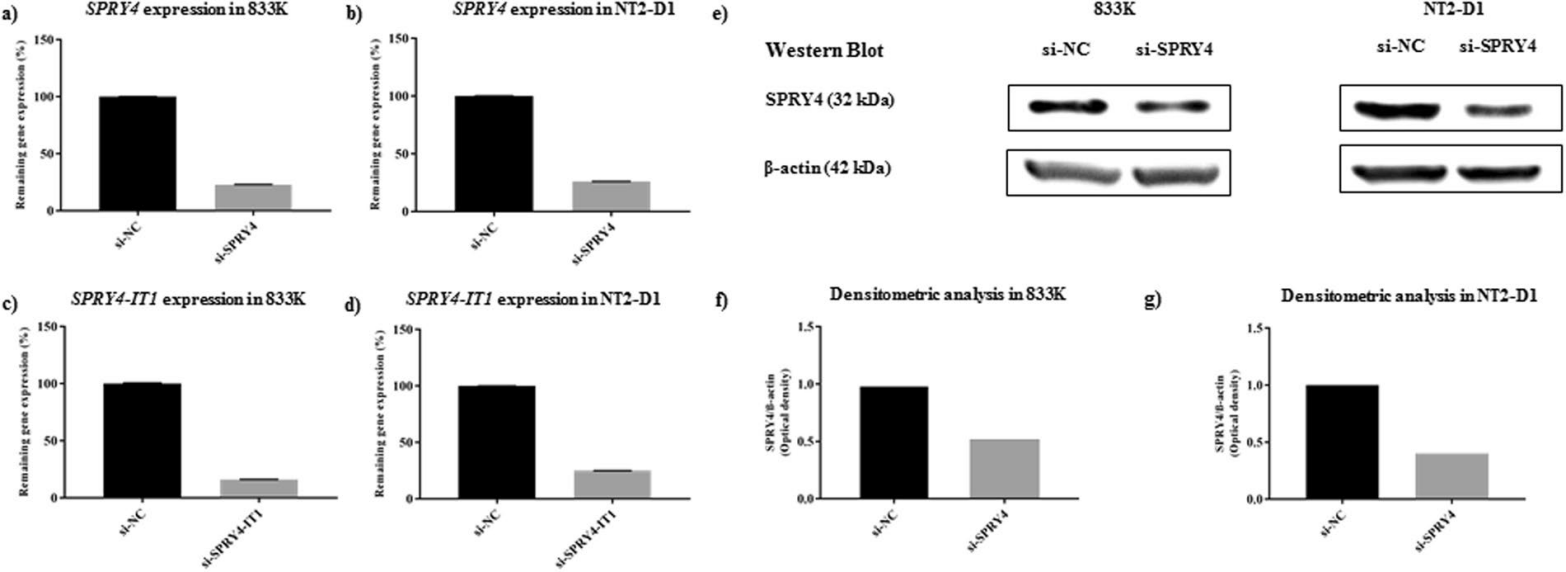
siRNA-mediated knockdown of *SPRY4* and *SPRY4-IT1*. Knockdown of *SPRY4* and *SPRY4-IT1* resulted in an average of 75% reduction in RNA expression levels (analysed by qPCR) in both cell lines 833 K (**a**,**c**) and NT2-D1 (**b**,**d**). As a vehicle control, a non-targeting negative control siRNA (si-NC) with the same chemical modifications was used. Western blot also showed a considerable reduction of SPRY4 protein expression in both cell lines after the knockdown (**e**). Densitometric analysis of the western blots shows that knockdown of SPRY4 protein was more efficient in NT2-D1 cells (**g**) than in 833 K cells (**f**) OD of SPRY4 was normalized to OD of β-actin. The cropped blots are used in the figure, and full-length blots are presented in Supplementary Fig. S2. The experiments were repeated at least three times, and a representative experiment is shown. *t*-test: Control vs siRNA, mean ± SD, statistical significance p < 0.05.

*SPRY4* and *SPRY4-IT1* were expressed in both NT2-D1 and 833 K cells. siRNA-mediated gene silencing resulted in an average of 75% reduction in *SPRY4* and *SPRY4-IT1* RNA expression levels in 833 K cells (Fig. 2a,c) and NT2-D1 cells (Fig. 2b,d). SPRY4 protein expression was also substantially decreased in both cell lines after knockdown (Fig. 2e), and densitometric analysis of the western blot shows that knockdown of SPRY4 protein was more efficient in NT2-D1 cells (Fig. 2f) than in 833 K cells (Fig. 2g).

### Effect of knockdown of *SPRY4* and *SPRY4-IT1* on cell growth, migration, and invasion

**Figure 3:**
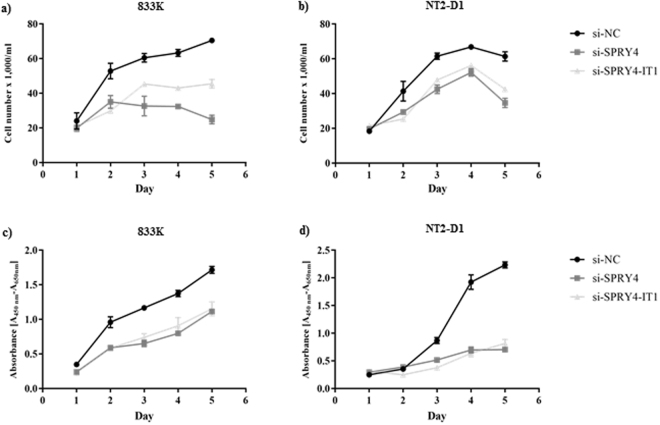
Cell counting and proliferation after knockdown of *SPRY4* and *SPRY4-IT1*. Knockdown of both *SPRY4* and *SPRY4-IT1* decreased the number of viable cells and the cell proliferation significantly both in 833 K (**a**,**c**) and NT2-D1 cells (**b**,**d**) in a time-dependent manner. The experiments were repeated at least three times, and a representative experiment is shown here. *t*-test: Control vs siRNA, mean ± SD, statistical significance p < 0.05.

**Figure 4:**
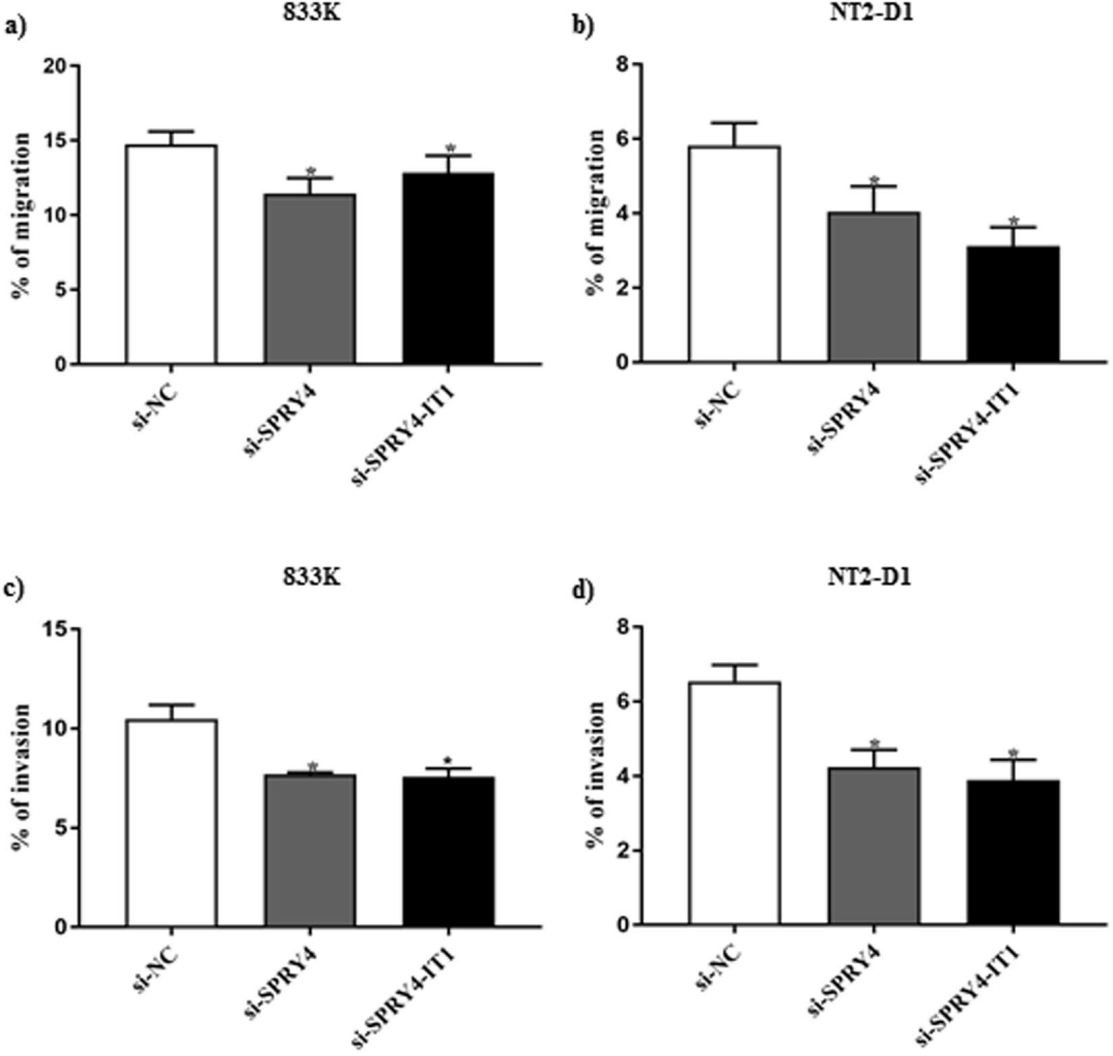
Cell migration and invasion after knockdown of *SPRY4* and *SPRY4-IT1*. Knockdown of both *SPRY4* and *SPRY4-IT1* reduced the cell migration and cell invasion both in 833 K (**a**,**c**) and NT2-D1 (**b**,**d**) cells respectively. Percentage of cell migration and invasion was analysed by converting relative fluorescence units (RFU) into a number of cells. The experiments were repeated at least three times, and a representative experiment is shown here. *t*-test: Control vs siRNA, mean ± SD, statistical significance p < 0.05.

Both *SPRY4* and *SPRY4-IT1* have been observed to alter cell growth, migration and invasion in prostate cancer and melanoma^20,22^. To investigate the effects of *SPRY4* and *SPRY4-IT1* knockdown on TGCT cell growth, we performed cell counting and cell proliferation assay after the knockdown of *SPRY4* and *SPRY4-IT1* in the 833 K and NT2-D1 cells lines. Knockdown of both genes led to a significant decrease in viable cell number (Fig. 3a,b) and cell proliferation (Fig. 3c,d) in a time-dependent manner relative to control. Knockdown of *SPRY4* and *SPRY4-IT1* also resulted in a significant reduction in cell migration (Fig. 4a,b) and invasion (Fig. 4c,d).

### Effect of knockdown of *SPRY4* and *SPRY4-IT1* on MAPK / ERK and PI3K / Akt signalling

**Figure 5:**
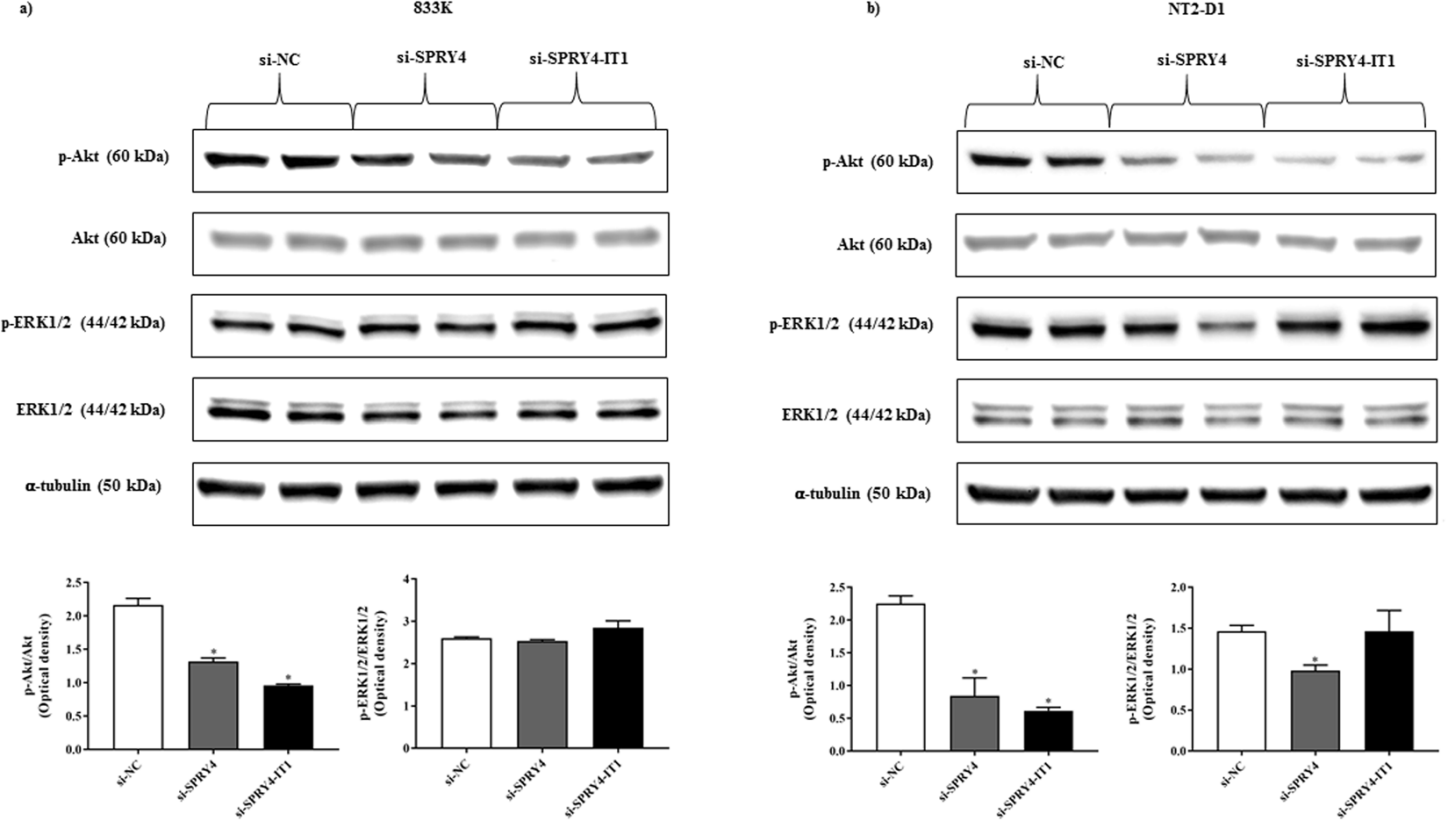
Effects of *SPRY4* and *SPRY4-IT1* knockdown on the activation of PI3/Akt and MAPK/ERK1/2 pathway. Western blot was performed to investigate the phosphorylation of Akt and ERK1/2 after knockdown of *SPRY4* and *SPRY4-IT1*. After knockdown of *SPRY4* and *SPRY4-IT1*, Akt phosphorylation was significantly inhibited in 833 K (**a**) and NT2-D1 (**b**) cells, particularly, inhibition of Akt phosphorylation was quite substantial in NT2-D1 cells by both *SPRY4* and *SPRY4-IT1* knockdown. However, phosphorylation of ERK1/2 only showed a weak but significant inhibition in NT2-D1 cells after the *SPRY4* knockdown. Akt and ERK1/2 were used as endogenous controls, and α-tubulin was used as a loading control. The bar graphs show the corresponding densitometric analyses of the western blots where the ratio of p-Akt/Akt, and the ratio of p-ERK1/2/ERK1/2 were calculated after normalising with α-tubulin. Samples were loaded as independent duplicates. The cropped blots are used in the figure, and full-length blots are presented in Supplementary Figs S3, 4. The experiments were repeated at least three times, and a representative experiment is shown. *t*-test: Control vs siRNA, mean ± SD, statistical significance p < 0.05.

Since increased cell proliferation in malignancies happens through the aberrant activation of MAPK / ERK and PI3K / Akt signalling pathways^17^, we investigated whether knockdown of *SPRY4* and *SPRY4-IT1* altered the activation of MAPK / ERK and PI3/Akt pathways. Knockdown of *SPRY4* and *SPRY4-IT1* significantly inhibited the phosphorylation of Akt in both 833 K and NT2-D1 cells, while phosphorylation of ERK1/2 showed a weak but significant inhibition in NT2-D1 cells only after *SPRY4* knockdown (Fig. 5a,b). We also examined the phosphorylation of Akt in the tissue and found phospho-Akt in all the TGCTs (Supplementary Fig. S5). A weak band was detected in one of the normal samples of comparable intensity with the one in moderately differentiated seminoma.

## Discussion

In our study, the finding of high expression of *SPRY4* and *SPRY4-IT1* in TGCTs compared with normal adult testis strengthens the hypothesis of a role of the *SPRY4* gene in TGCT development. The distinct difference in expression between TGCT and normal tissue was also observed for the SPRY4 protein. Particularly, no detection of SPRY4 protein in any normal testis sample, and the profound difference of SPRY4 protein expression between moderately differentiated seminoma sample and undifferentiated seminoma sample indicate that *SPRY4* may act as an oncogene in TGCT pathogenesis. We found lower RNA expression levels of *SPRY4* and *SPRY4-IT1* in seminoma than in other TGCT subtypes but had no information about the status of differentiation of these two seminoma samples. The only report of higher expression of *SPRY4* and *SPRY4-IT1* in malignant tissue than in the normal is from human melanoma tissue relative to melanocytes^22^. Genetic knockdown of *SPRY4* and *SPRY4-IT1* in 833 K and NT2-D1 cells resulted in a decrease in cell survival, proliferation, migration, and invasion. This is in accordance with a study in human ovarian cancer cells where suppression of *SPRY4* attenuated growth factor-induced cell migration and invasion^21^. Furthermore, suppression of *SPRY4-IT1* in human melanoma cells inhibited cell growth, migration, and invasion^22^, indicating oncogenic activity. However, an opposite role of *SPRY4* and *SPRY4-IT1* has been reported in other types of cancer. For example, SPRY4 showed tumour suppressor activity in lung^19^, prostate^20^, and breast cancer^28^, and *SPRY4-IT1* showed tumour suppressor activity in lung cancer^25^.

In malignancies, the role of *SPRY* gene family on RTK-mediated MAPK / ERK and PI3K / Akt signalling seems to be cell-specific and context-dependent^18^. In our study, silencing of *SPRY4* and *SPRY4-IT1* in both the TGCT cell lines displayed a potent inhibition of Akt phosphorylation. Phospho-Akt was detected in all the TGCTs, but only in one normal testis sample. Furthermore, the level was low in both the normal sample and the moderately differentiated seminoma. These findings support that activation of PI3K / Akt signalling plays a role in TGCT development, as also reported in another study^29^. Furthermore, *SPRY4* knockdown in NT2-D1 cells resulted in a weak but significant inhibition of ERK1/2 phosphorylation. The degree of *SPRY4* knockdown attained in the 833 K cells might not be sufficient to observe an inhibitory effect on phosphorylation of ERK1/2. To our knowledge, this is the first study to show an inhibitory effect of *SPRY4* and *SPRY4-IT1* knockdown on RTK signalling in any cancer cell type. In contrast to our findings, suppressing *SPRY4* in human breast carcinoma cells resulted in a substantial increase in phosphorylation of Akt and a mild increase in phosphorylation of ERK1/2^28^. Interestingly, the regulatory function of *SPRY4* on RTK signalling has been shown to be ligand-specific. In human embryonic kidney fibroblasts and mouse embryonic fibroblasts, *SPRY4* decreased the FGF-induced activation of ERK1/2 but increased the EGF-induced ERK1/2 activation^30,31^. As far as we know, the effect of *SPRY4-IT1* on RTK signalling in any other cell type has not been reported.

Protein-coding genes and their associated intronic lncRNAs usually display positively correlated expression profiles^32,33^. Khaitan *et al*. reported a positive correlation between the expressions of *SPRY4* and *SPRY4-IT1* across different normal tissues and melanoma patient tissues. In normal tissue, *SPRY4-IT1* showed higher expression than *SPRY4* and also larger variation. In the melanoma samples, however, the expression level of *SPRY4* was higher than that of *SPRY4-IT1* in some samples and vice versa in others^22^. In line with this, we found higher expression of *SPRY4* than that of *SPRY4-IT1* in three of the TGCT subtypes, whereas, in teratoma, the expression of *SPRY4-IT1* was higher than that of *SPRY4*. The higher levels of the host gene *SPRY4* and its intronic lncRNA *SPRY4-*IT1 with variable expression patterns in different TGCT subtypes suggest they are not simply oncogenes but rather have some independent regulatory mechanisms. In a follow-up study by Khaitan’s group, Mazar *et al*. showed that genetic knockdown of neither *SPRY4* nor *SPRY4-IT1* altered the expression of the other and that *SPRY4-IT1* displayed stronger response to growth factors compared to *SPRY4*. Furthermore, they observed that *SPRY4* and *SPRY4-IT1* transcript decay was independently regulated. They also found that human melanoma cell invasion was 50% reduced by *SPRY4-IT1* knockdown but unaffected by the *SPRY4* knockdown and that *SPRY4-IT1* silencing induced apoptosis more effectively than did *SPRY4* knockdown^34^. These findings indicate both transcriptional and functional independence of the host gene *SPRY4* and its lncRNA *SPRY4-IT1*. However, in our study, we showed similar effects of *SPRY4* and *SPRY4-IT1*.

A challenge in performing expression studies in TGCTs is the limited availability of healthy adult testis tissue to be used as a control. Researchers often use tissue adjacent to tumour tissue as a normal control, which may have cancer-associated genetic characteristics and raises questions^35^. The use of normal testis samples is a strength in our study. Results from *in vitro* studies may not be the representative of the situation *in vivo*. However, there is no appropriate *in vivo* model for human TGCT, and no animal model has so far been able to form the precursor *carcinoma in situ* cells observed in human^36^. The cell lines NT2-D1 and 833 K used in our study were derived from human metastatic TGCT tissues of lung and abdomen, respectively. Both TGCT cell lines exhibit characteristics similar to EC cells, which represent the pluripotent stem cells of teratocarcinomas^37^. NT2-D1 cells also have common characteristics with teratoma and 833 K cells with teratoma, yolk sac tumour, and seminoma^26,27^. EC cell lines have received the most attention as an experimental model for functional studies of TGCT in lack of suitable animal models^37^.

**Figure 6:**
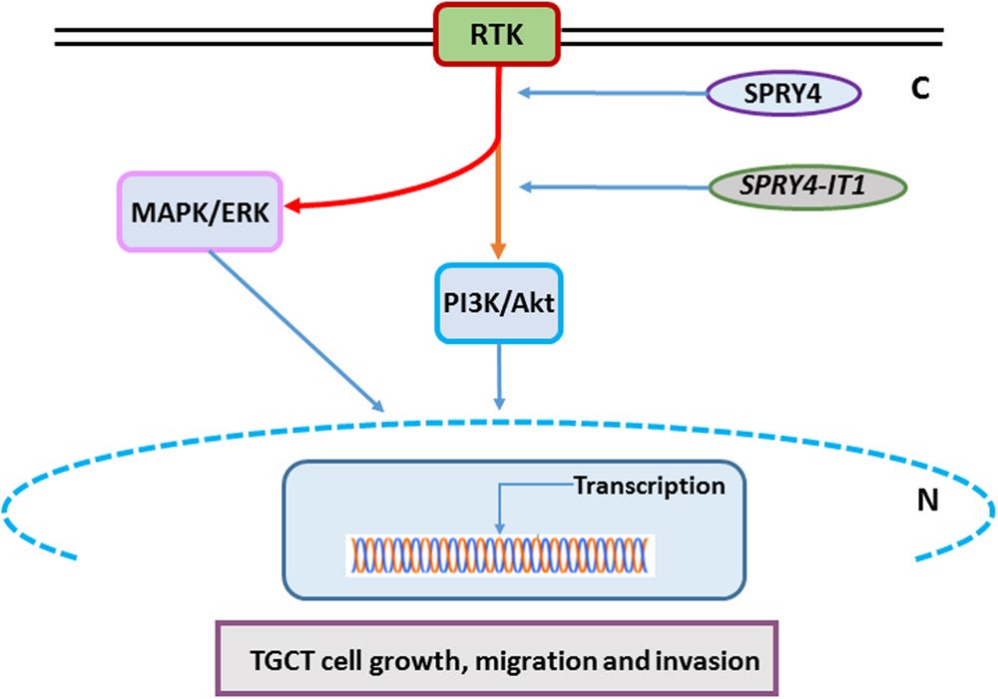
A model for MAPK/ERK and PI3K/Akt activation of SPRY4 and *SPRY4-IT1* in TGCT cells. Knockdown of *SPRY4* and *SPRY4-IT1* results in decreased cell growth, migration, and invasion.

Our results suggest that knockdown of both *SPRY4* and *SPRY4-IT1* inhibit TGCT growth by inhibiting the activation of PI3K / Akt pathway (Fig. 6), thus, acting as oncogenes. Further mechanistic studies of *SPRY4* and *SPRY4-IT1* are needed to get more knowledge about TGCT pathogenesis. Studies of the interaction with proteins encoded by other susceptibility genes, such as *KITLG*, *BAK1* and *DMRT1*, may also advance the understanding of the mechanisms behind TGCT development.

## Methods

### Tissue

For the expression analysis, 13 TCGT samples of different patients were provided by Dr Rolf I. Skotheim (Genome Biology Group, Oslo University Hospital), consisting of yolk sac tumour (n = 3), embryonal carcinoma (n = 4), teratoma (n = 3), seminoma (n = 2), and choriocarcinoma (1) subtypes^38^. Normal testicular tissue samples were collected from 11 adult organ transplant donors with no known history of cancer. For immunoblotting, a series of protein lysates made from TGCT tissues was bought from Protein Biotechnologies (CA, USA). The study has been approved by the Regional Committee for Medical and Health Research Ethics, Norway (2016 / 2006, REC South‐East), and all experiments were performed in accordance with approved guidelines and regulations. Donors of the TGCT samples provided informed written consent, and for the normal testis samples in connection with organ transplantation, consent was obtained according to the Norwegian legislation relating to transplantation, hospital autopsies and the donation of bodies.

### Cell culture

Two TGCT cell lines, NT2-D1 and 833 K were kindly provided by Dr Birgitte Lindeman (Norwegian Institute of Public Health, Oslo). 833 K and NT2-D1 were cultured in RPMI-1640 and DMEM medium, respectively, supplemented with 10% foetal bovine serum at 37 °C in a humidified 5% CO_2_ incubator. The morphology of the cells was regularly checked, and stocks of cell lines were passaged not more than ten times for use in experiments.

### Knockdown experiments

To knockdown the expression of *SPRY4* and *SPRY4-IT1* in 833 K and NT2-D1 cells, small-interfering RNA (siRNA) based gene silencing technology was applied. siRNA transfection protocol was adopted from Felfly *et al*. with slight modification^39^. Cells were seeded out in a 6-well plate and grown overnight. Lipofectamine RNAiMAX (Invitrogen, CA, USA) transfection mix was prepared with siRNA sets (Supplementary Table S1) and applied to 833 K and NT2-D1 cells to knockdown the expression of *SPRY4* and *SPRY4-IT1*. 100% transfection efficiency was confirmed by using a plasmid encoding green fluorescent protein. The expression of this protein in transfected cells was detected by fluorescence microscopy (data not shown). After 48 hours of transfection, cells were harvested and stored at −70 °C until further use. Knockdown was verified using qPCR and western blot.

### Quantitative PCR (qPCR)

RNA from cell lines and tissue samples were extracted using RNeasy (Qiagen, CA, USA), and 100 ng of RNA was converted to cDNA using TaqMan Reverse Transcription Reagents Kit (Applied Biosystems, CA, USA). qPCR was performed using 0.5 ng of cDNA and TaqMan Pre-Developed Assay Reagents (Applied Biosystems, CA, USA) under recommended conditions on a Mx3005P instrument (Agilent Technologies, Santa Clara, USA). All samples were run in triplicates, and the relative expression was calculated using the equation RQ = 2^−ΔΔCT^. CT values > 35 were regarded as negative. *RPS29* has been shown to be stably expressed in adult human testis and germ cell neoplasms^40^ and was used as a reference gene in our study. The primers used are listed in Supplementary Table S2.

### Western blot

Proteins were isolated after transfection with siRNAs using RIPA buffer (SIGMA-ALDRICH) containing 150 mM NaCl, 1.0% IGEPAL® CA-630, 0.5% sodium deoxycholate, 0.1% SDS, 50 mM Tris, pH 8.0, phosphatase inhibitors and protease inhibitors. The protein concentration was measured using protein assay dye reagent concentrate (BioRad), and 30 µg protein was loaded onto 10% Mini-PROTEAN® TGX™ Precast Gels (Bio-rad), unless otherwise specified (Supplementary Fig. S5). After SDS-PAGE, the proteins were blotted onto a PVDF membrane, and the membrane was blocked in TBST with 5% skim milk before incubating with primary antibody overnight at 4 °C. An HRP conjugated secondary antibody was used, and the proteins were detected using the ImageLab machine (BioRad). Optical density (OD) of the protein bands was determined by using Image Studio Lite. Primary antibodies used in this study were: Anti-β-actin (Abcam, 1:1000), Anti-phospho-ERK1/2 (Cell Signaling, 1:2000), Anti-ERK1/2 (Cell Signaling, 1:1000), Anti-phospho-Akt (ser473) (Cell Signaling, 1:2000), Anti-Akt (Cell Signaling, 1:1000), Anti-SPRY4 (Abcam, 1:1000). HRP-conjugated anti-rabbit (Cell Signaling, 1:2500) was used as secondary antibody.

### Cell counting

Cells (120000) were seeded out in 6-well plates and grown overnight. After 48 hours of transfection with siRNAs, viable cells were counted during a period of five days by use of a haemocytometer. The cells were stained with trypan blue before counting to exclude dead cells.

### Cell proliferation

The proliferative capacity of the cells was examined by XTT assay (Roche). After 48 hours of transfection with siRNAs, cells were seeded out in a 96-well plate and cultured at a density of 3 × 10^3^ cells/well. After a period of five days’ incubation, the cells were treated with 50 ul of XTT solution. The absorbance was measured at 450 nm with a microplate reader after 24 hours of incubation.

### Cell migration and invasion

For the cell migration and invasion assays, cells were seeded out and cultured overnight, followed by siRNAs transfection and serum-deprivation for 24 hours. The cells were then harvested and assayed in a 96-well Boyden Chamber (R&D Systems) for migration and invasion according to manufacturer’s protocol.

### Statistical analysis

The results were analysed by *t*-test using the PRISM software. Significant differences were defined by P-values < 0.05.

### Data availability statement

The datasets generated during and/or analysed during the current study are available from the corresponding author on reasonable request.

## Electronic supplementary material

Dataset 1
