# Supplementary material for: Knockdown of SPRY4 and SPRY4-IT1 inhibits cell growth and phosphorylation of Akt in human testicular germ cell tumours
Source: Sci Rep. 2018 Feb 6;8:2462. doi: 10.1038/s41598-018-20846-8 (PMC5802735; doi:10.1038/s41598-018-20846-8)
Supplement: Supplementary file 1 — Dataset 1 [file 41598_2018_20846_MOESM1_ESM.doc]

# Supplementary Information

# for

# Knockdown of *SPRY4* and *SPRY4-IT1* inhibits cell growth and phosphorylation of Akt in human testicular germ cell tumours

Mrinal K. Das^1*^, Kari Furu^1,2^, Herman F. Evensen^1^, Øyvind P. Haugen^1,3^ & Trine B. Haugen^1^

# ^1^Faculty of Health Sciences, OsloMet – Oslo Metropolitan University, Oslo, Norway.

# ^2^Cancer Registry, Oslo, Norway

# ^3^Faculty of Dentistry, University of Oslo, Oslo, Norway

* Correspondence and requests for materials should be addressed to M.K.D (email: Mrinal-kumar.das@hioa.no)

## Supplementary information includes:

1. Supplementary Table S1
2. Supplementary Table S2
3. Supplementary Figure S1
4. Supplementary Figure S2
5. Supplementary Figure S3
6. Supplementary Figure S4
7. Supplementary Figure S5

# Supplementary Table S*1*: List of siRNAs

| **siRNA ID** | **Name** | **Distributor** | **Sequence** |
| --- | --- | --- | --- |
|  | Silencer® Select Negative Control No. 1 siRNA | Ambion, Applied Biosytems | Not provided |
| s37824 | Silencer Select Pre-designed SPRY4 | Ambion, Applied Biosystems | GCACGUCCUCAUUCGUGCag |
| s37825 | Silencer Select Pre-designed SPRY4 | Ambion, Applied Biosystems | CAACGCUCUUAGACCACAtt |
| s37826 | Silencer Select Pre-designed SPRY4 | Ambion,  Applied Biosystems | UGUGGAGAAUGACUACAUAtt |
|  | Customized Stealth siRNA SPRY4 | Invitrogen,  Applied Biosystems | GAGGCCUGUGGGAAGUGUAAAUGCA |
|  | Customized Stealth siRNA SPRY4-IT1 | Invitrogen,  Applied Biosystems | UUAAUAGGCCUUGGAAUC AGAAAGC |

# Supplementary Table S*2*: List of TaqMan gene expression assays

| **Gene name** | **Gene symbol** | **TaqMan assay ID** | **Distributor** | **Sequence** |
| --- | --- | --- | --- | --- |
| *Sprouty 4* | *SPRY4* | Hs01935412_s1 | Thermofisher Scientific | Not provided |
| *Sprouty 4 intronic transcript 1* | *SPRY4-IT1* | Hs03865501_s1 | Thermofisher Scientific | Not provided |
| *Ribosomal Protein S29* | *RPS29* | Hs03004310_g1 | Thermofisher Scientific | Not provided |

# Supplementary Figure S1


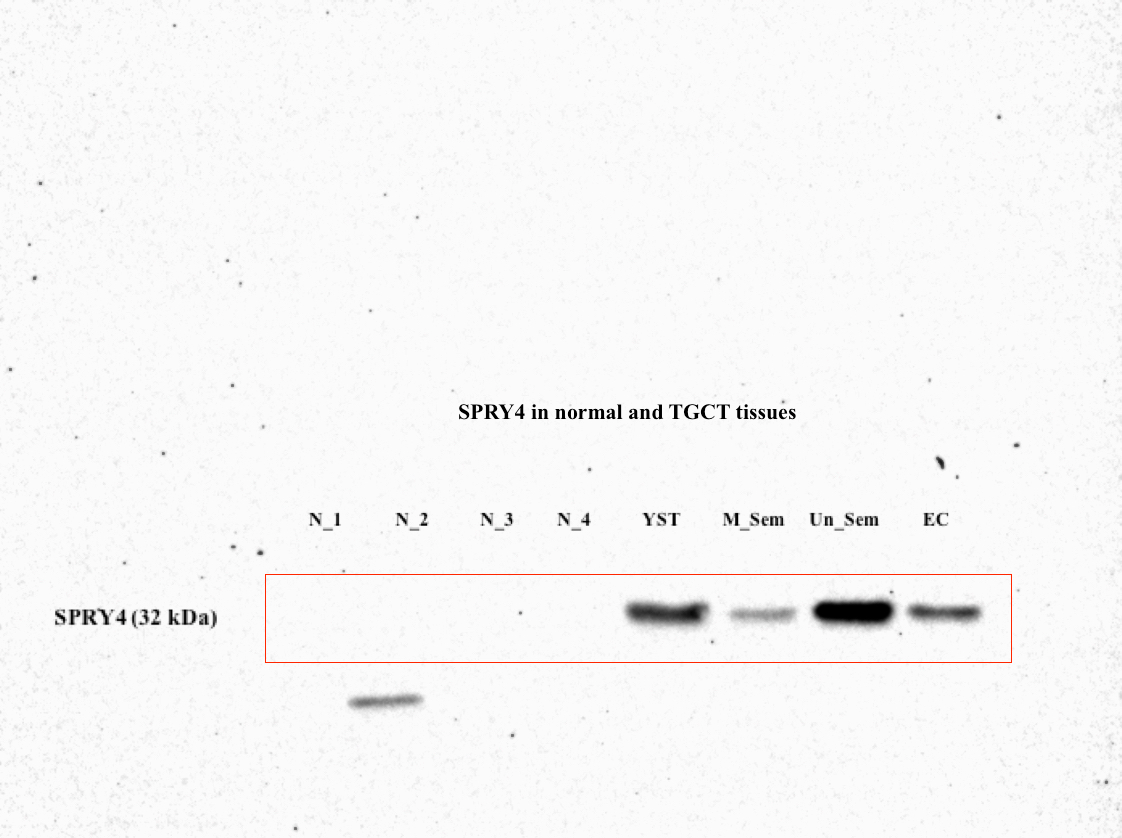


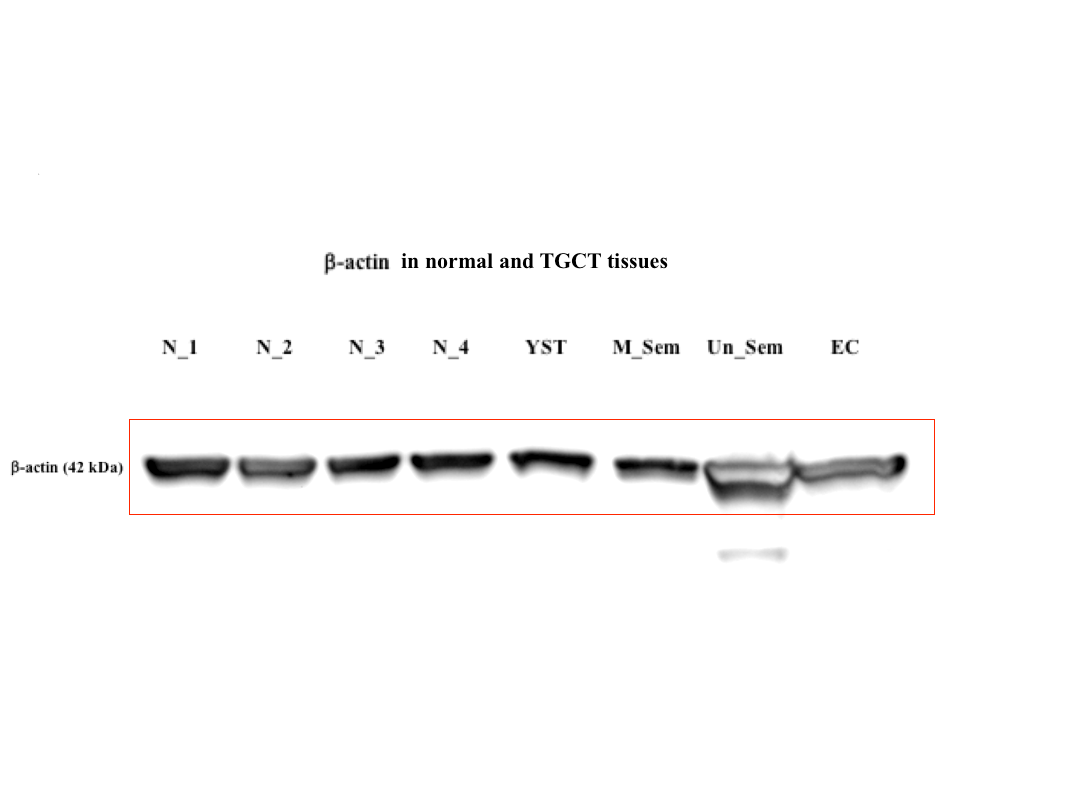


**Supplementary Figure S1**: Full length blots of Fig 1b. Red rectangle shows the cropping location.

# Supplementary Figure S2


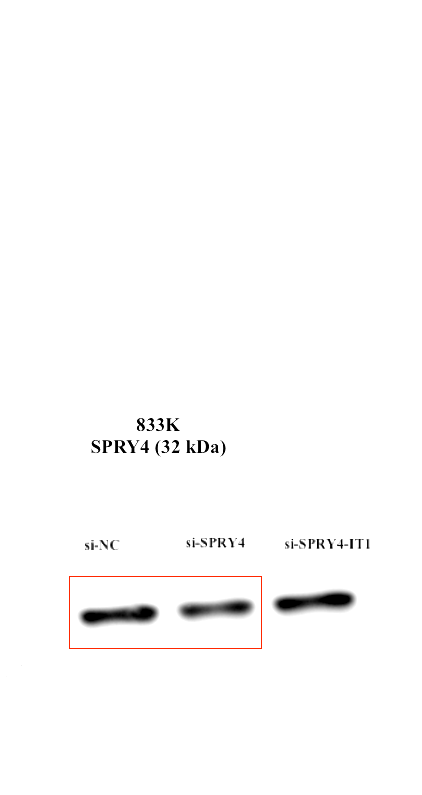

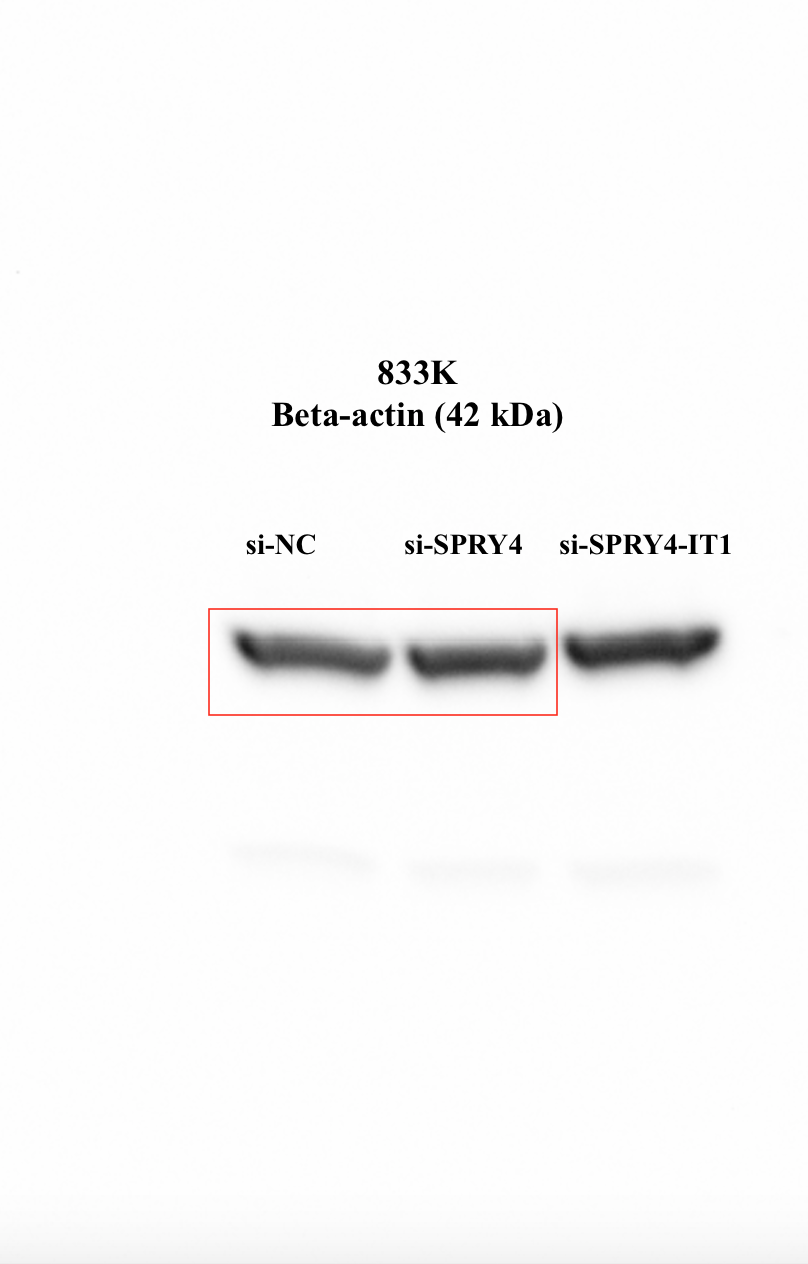


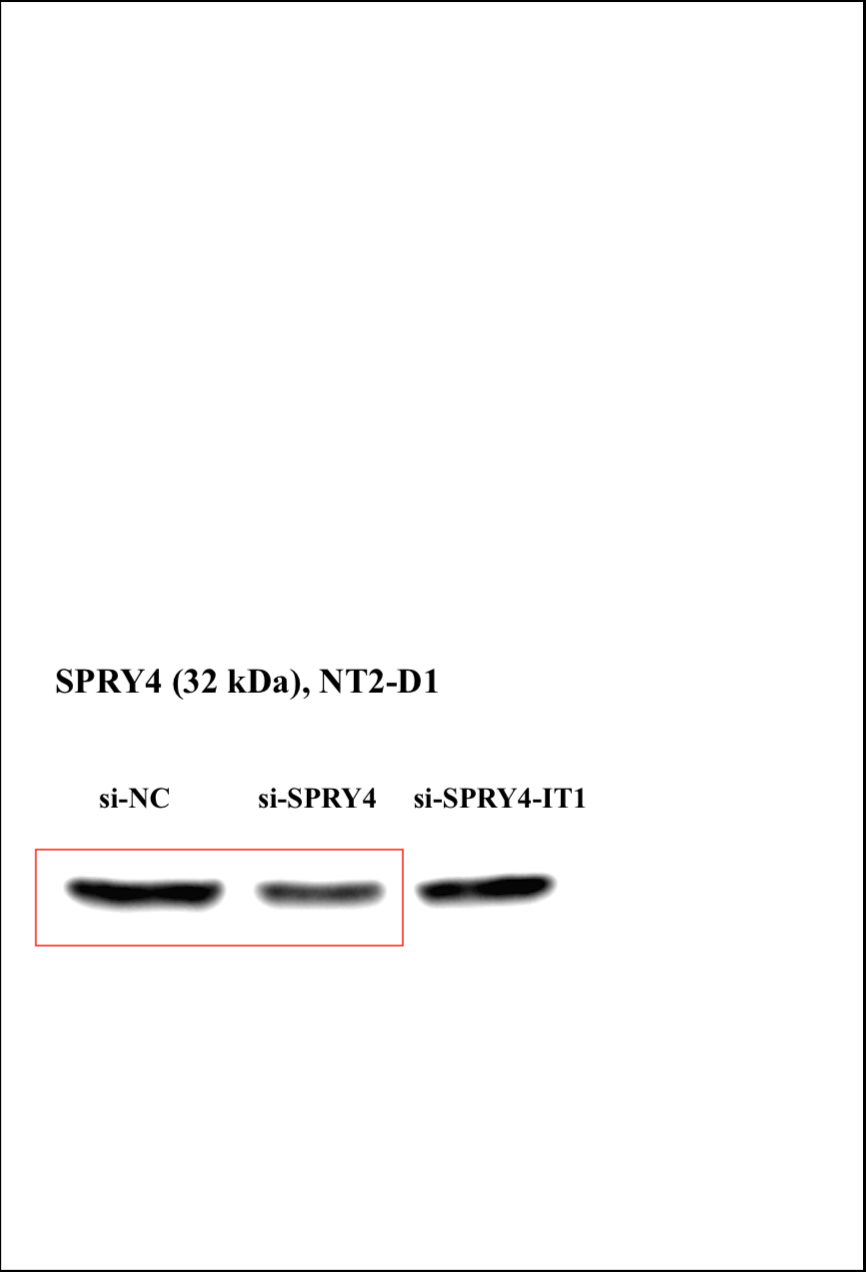

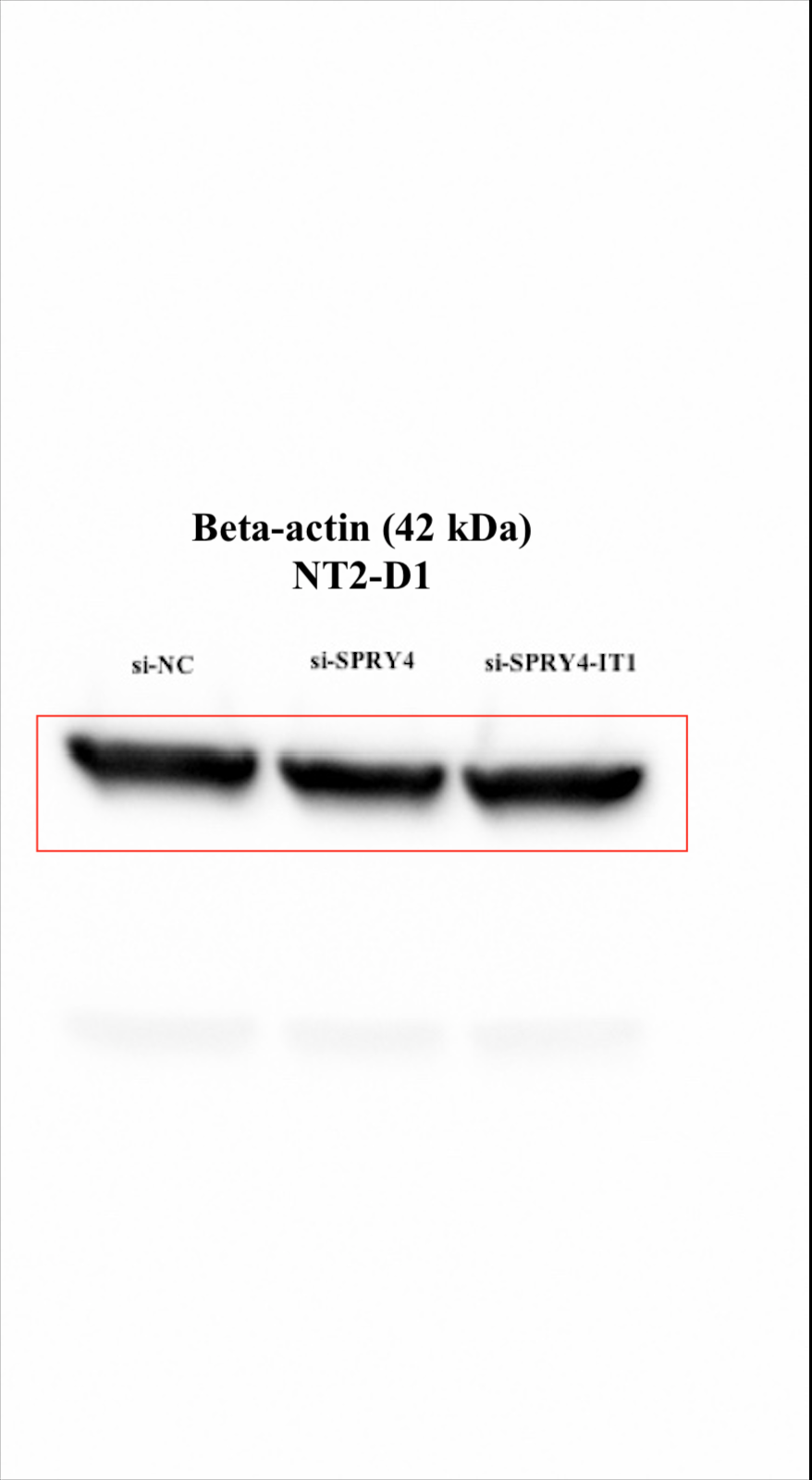


**Supplementary Figure S2**: Full length blots of Fig 2c. Red rectangle shows the cropping location.

# Supplementary Figure S3


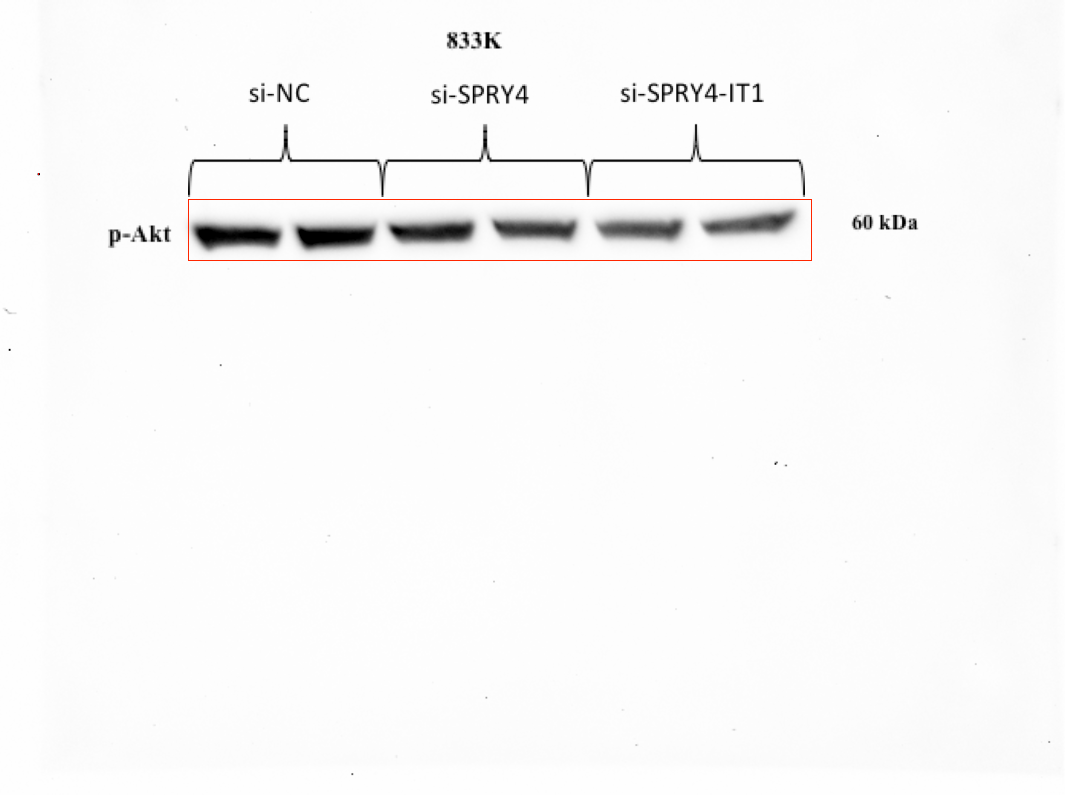

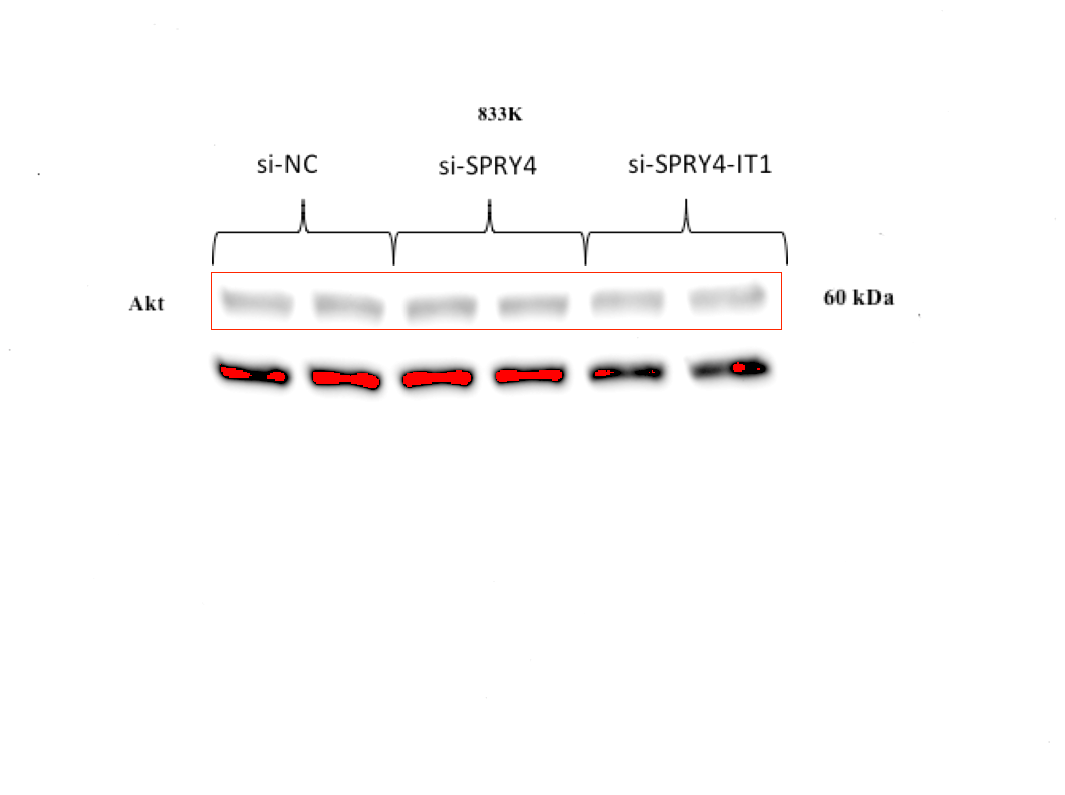


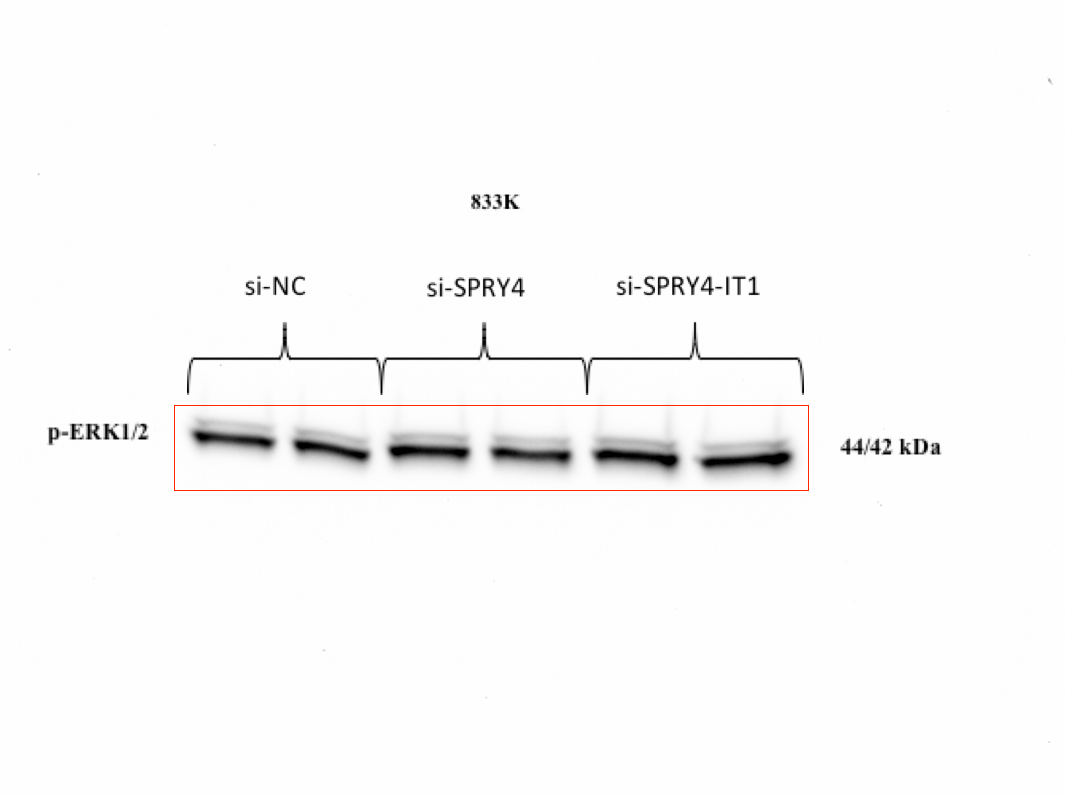

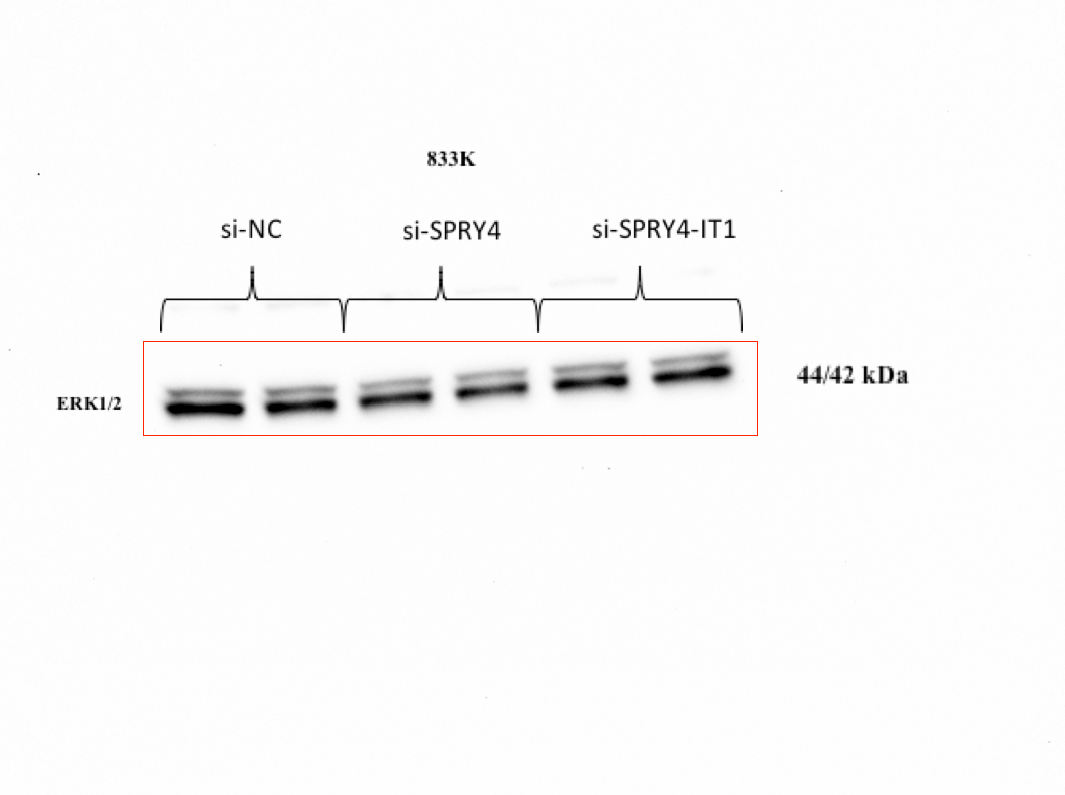


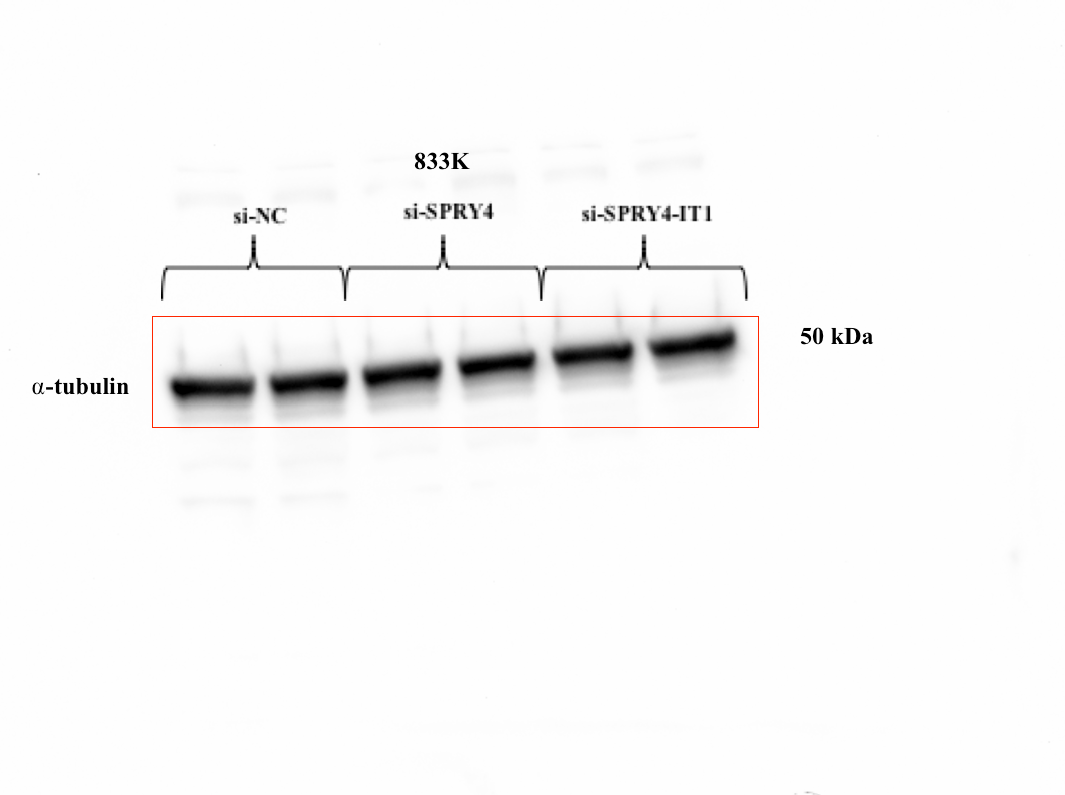


**Supplementary Figure S3**: Full length blots of Fig 5a. Red rectangle shows the cropping location.

# Supplementary Figure S4


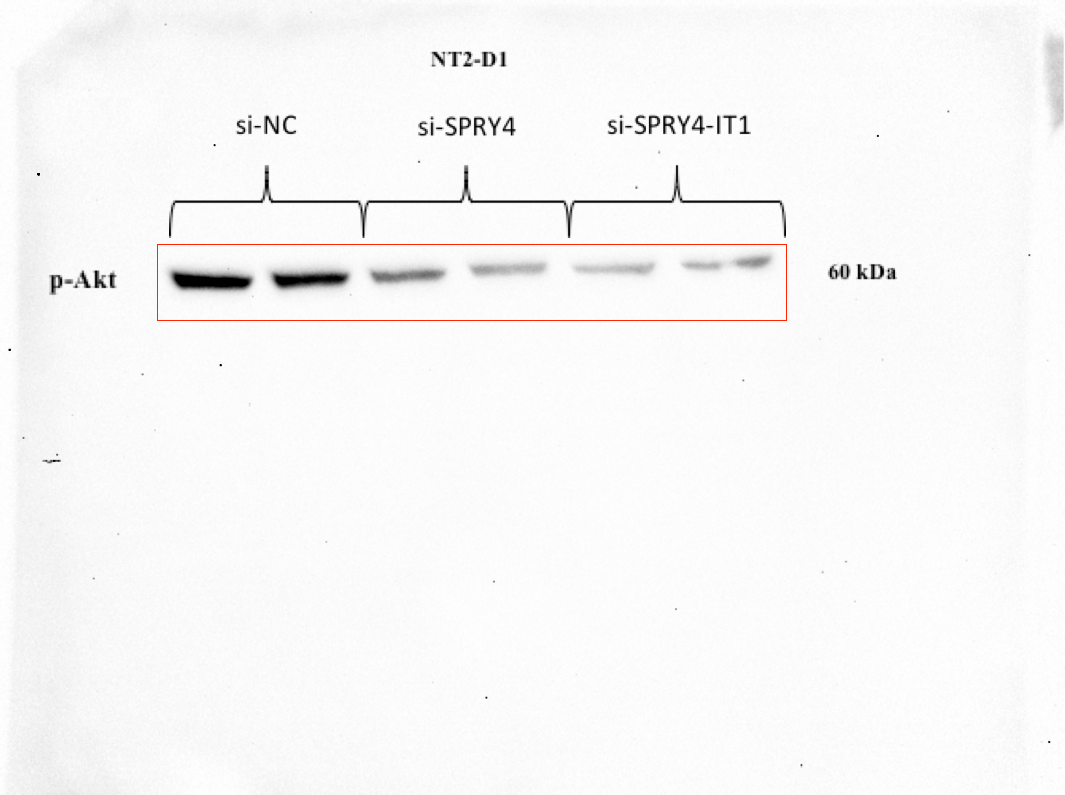

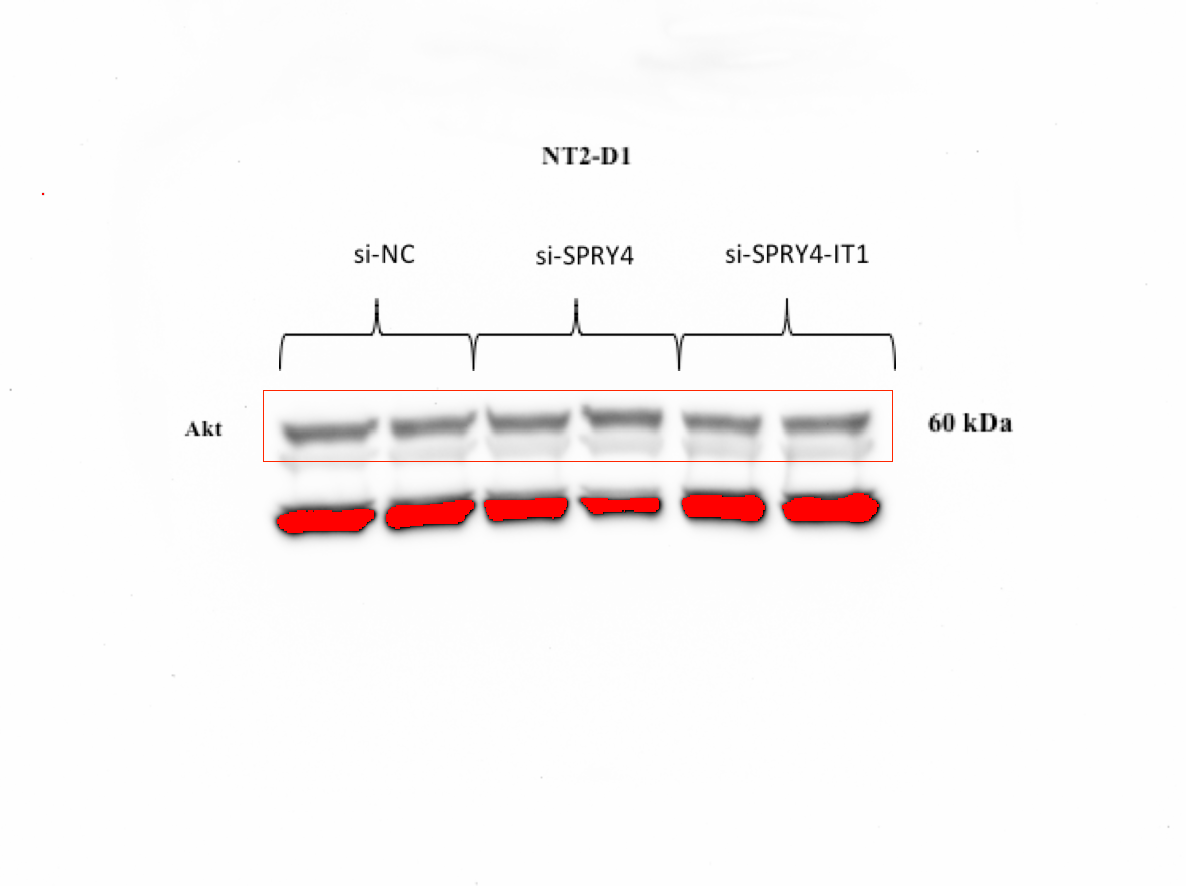


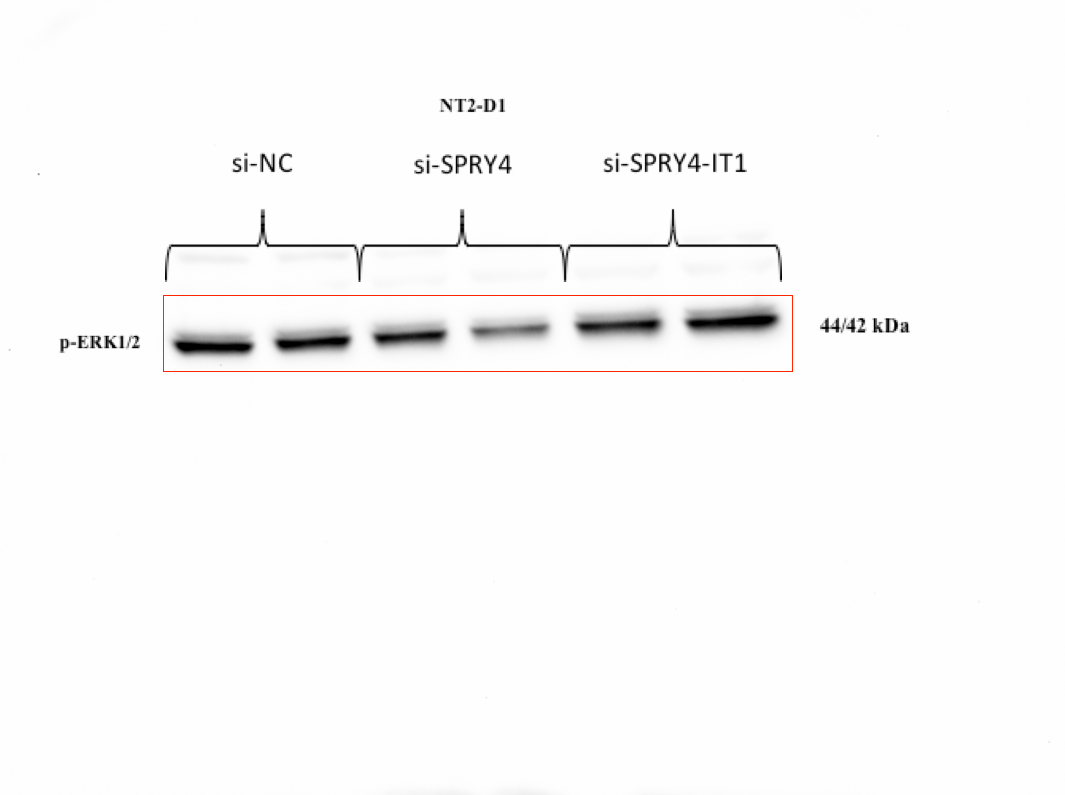

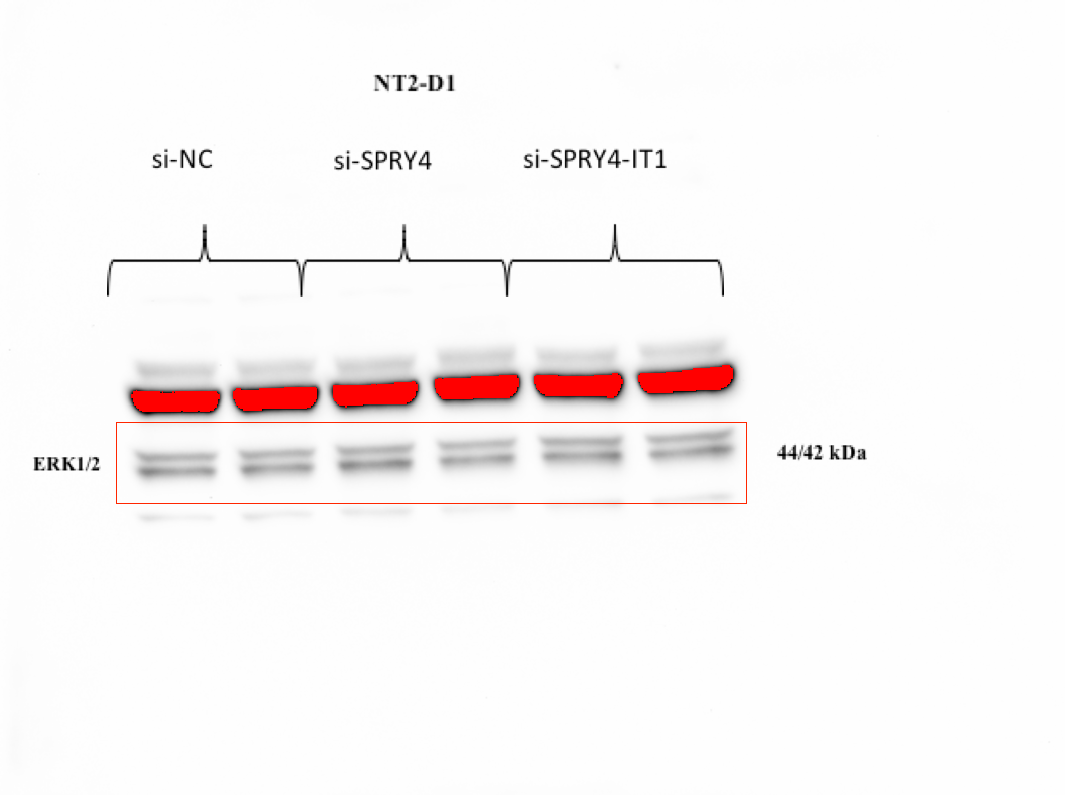


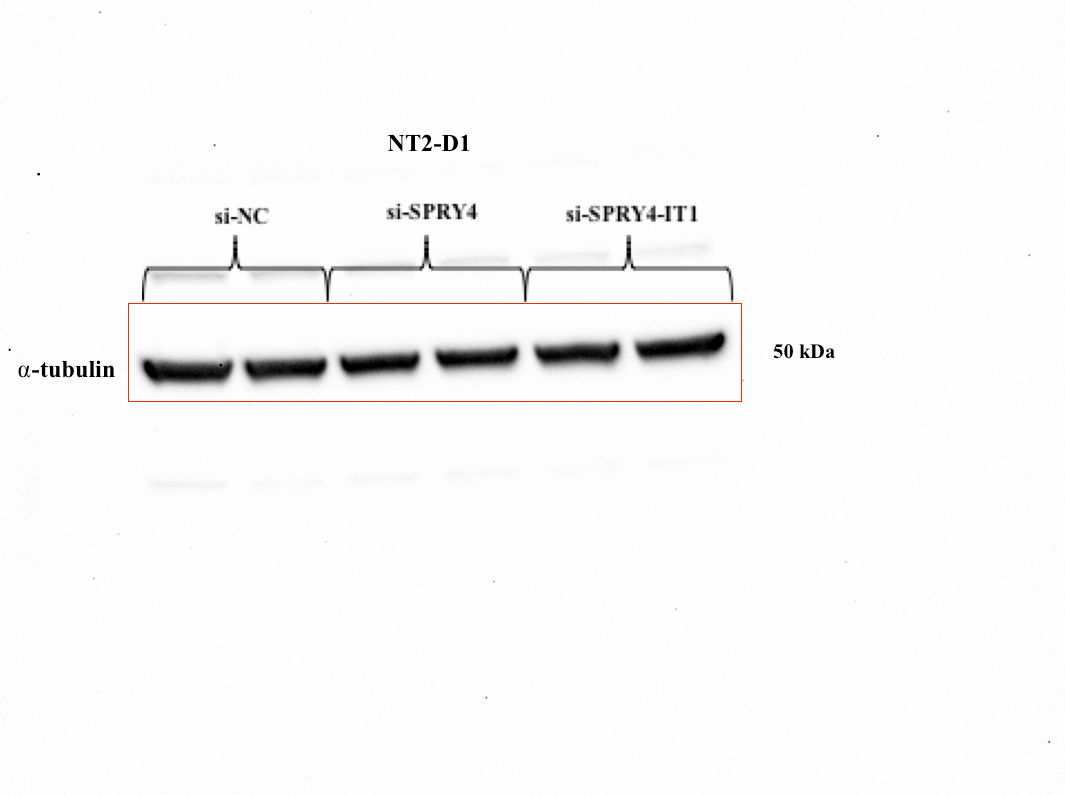


**Supplementary Figure S4**: Full length blots of Fig 5b. Red rectangle shows the cropping location.

# Supplementary Figure S5


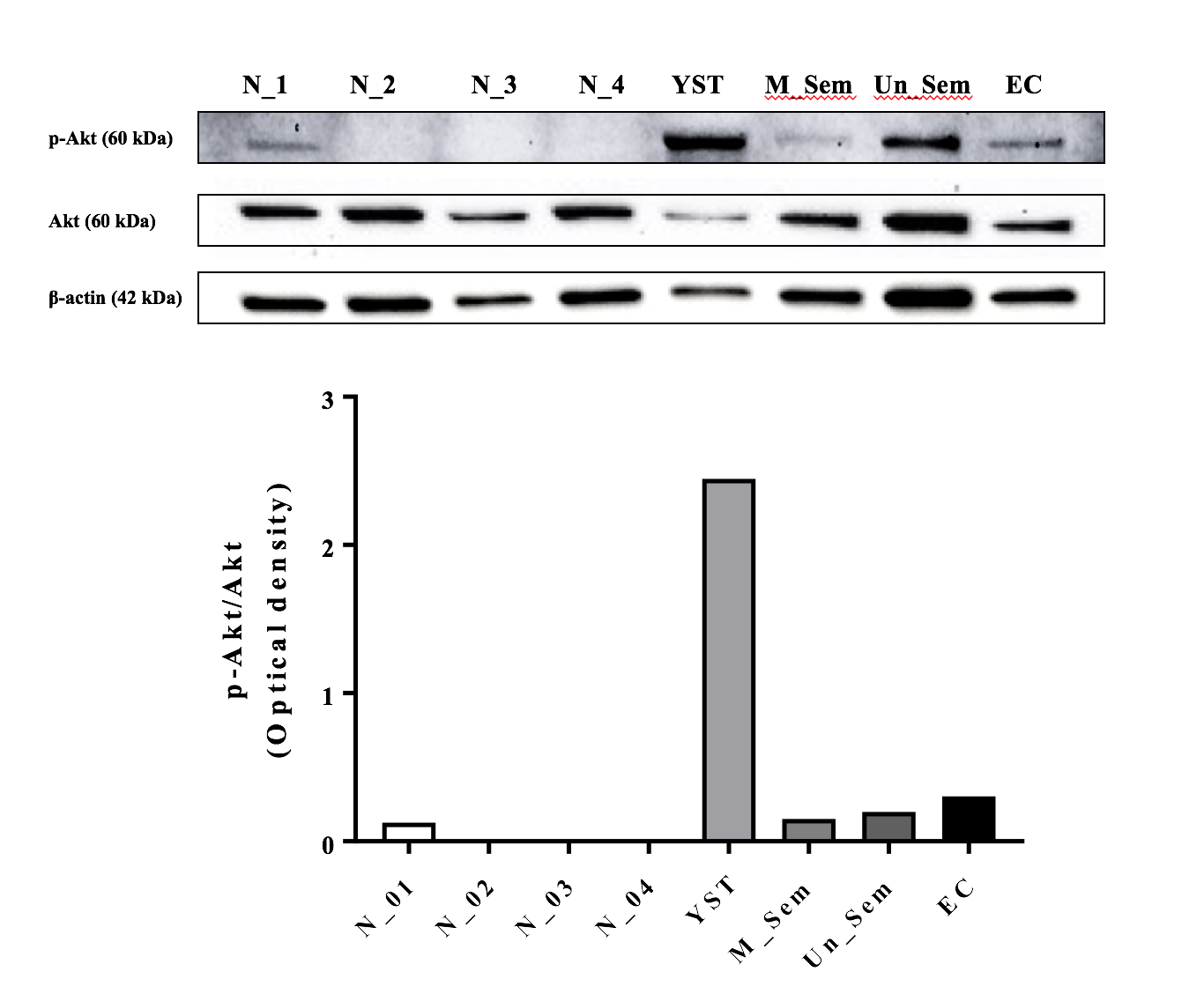


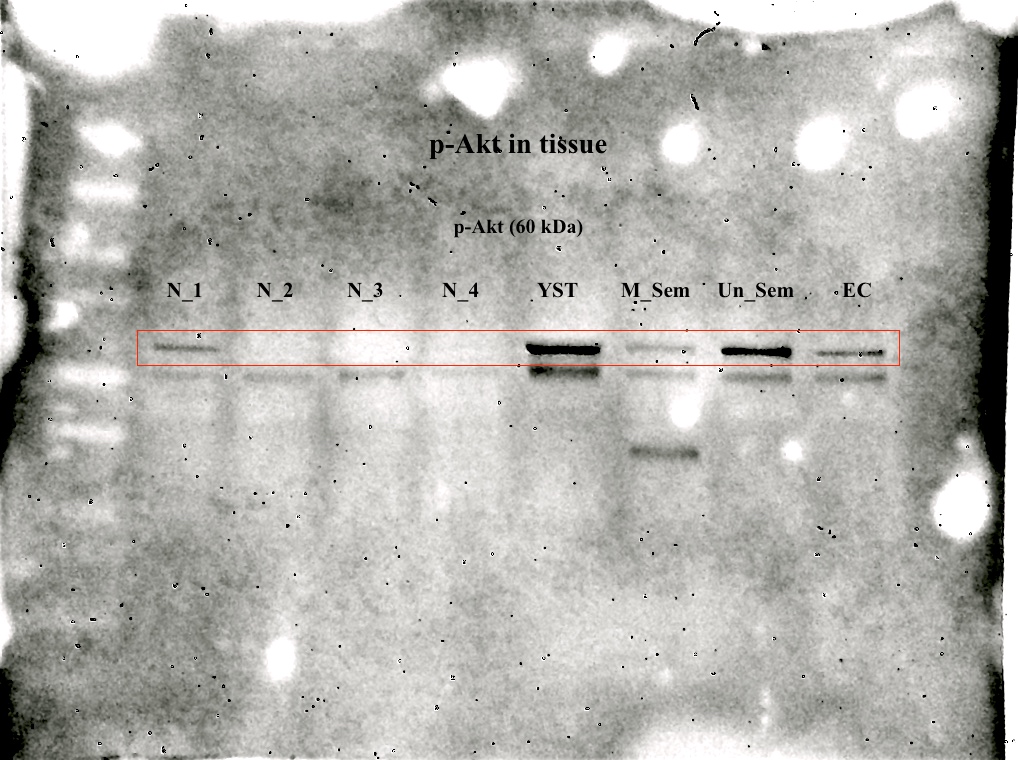

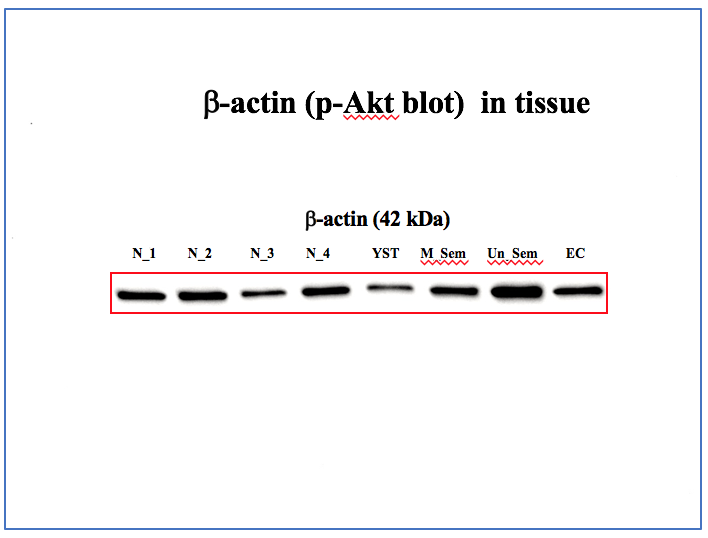


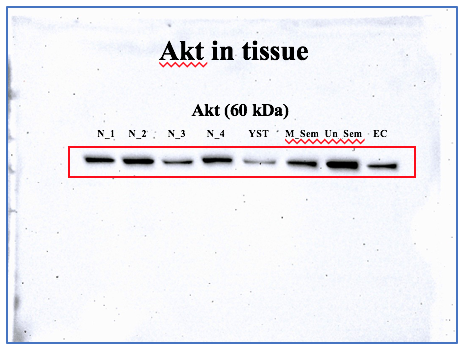

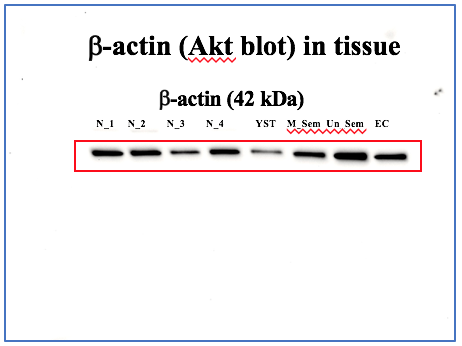


**Supplementary Figure S5**: Phosphorylation of Akt in TGCTs. The phospho-Akt was measured in TGCT and normal testis samples by western blot where 15 μg of protein was loaded for detection. p-Akt was detected in various amounts in the TGCT subtypes and in one of the normal testis sample, although low. The densitometric analysis of the western blots shows that the level of p-Akt was higher in YST, UN_Sem and EC compared to N_01. The ratio of p-Akt/Akt was calculated after normalization with β-actin. N (normal); YST (yolk sac carcinoma); M_Sem (moderately differentiated seminoma); Un_Sem (undifferentiated seminoma); EC (embryonal carcinoma). Full length blots are also presented and red rectangle shows the cropping location.
